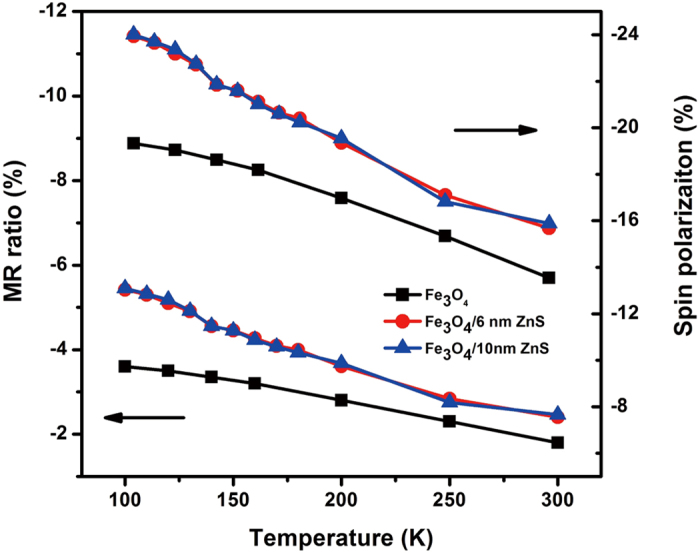# Investigation on Spin Dependent Transport Properties of Core-Shell Structural Fe_3_O_4_/ZnS Nanocomposites for Spintronic Application

**DOI:** 10.1038/srep11164

**Published:** 2015-06-08

**Authors:** Er Liu, Honglei Yuan, Zhaoxia Kou, Xiumei Wu, Qingyu Xu, Ya Zhai, Yunxia Sui, Biao You, Jun Du, Hongru Zhai

**Affiliations:** 1 Department of Physics, Southeast University, Nanjing 211189, China; 2 National Laboratory of Solid Microstructures, Nanjing University, Nanjing 210093, China; 3 Center for Material Analysis, Nanjing University, Nanjing 210093, China

## Abstract

The core-shell structural Fe_3_O_4_/ZnS nanocomposites with controllable shell thickness were well-fabricated via seed-mediate growth method. Structural and morphological characterizations reveal the direct deposition of crystalline II-VI compound semiconductor ZnS shell layer on Fe_3_O_4_ particles. Spin dependent electrical transport is studied on Fe_3_O_4_/ZnS nanocomposites with different shell thickness, and a large magnetoresistance (MR) ratio is observed under the magnetic field of 1.0 T at room temperature and 100 K for the compacted sample by Fe_3_O_4_/ZnS nanocomposites, which is 50% larger than that of sample with pure Fe_3_O_4_ particles, indicating that the enhanced MR is contributed from the spin injection between Fe_3_O_4_ and ZnS layer.

Magnetic/non-magnetic hetero-structured films have attracted growing interests due to their great developments in the field of spintronics^1^,^2^,^3^, including spin hall effect^4^ in magnetic/non-magnetic metallic bilayers, spin injection effect^5^,^6^ in magnetic tunnel junctions, spin transfer torque effect^7^ in magnetic spin valve and magnetic nano-oscillator^8^,^9^. Similar magnetic hetero-structured system, core shell structural magnetic nanocmposites combined magnetic core with non-magnetic shell layer is also a promising spintronics material, particular as their advantages of facile-fabrication and easy assembling. Thus the magnetic core shell structure has become a new research branch in spintronics extending from magnetic/amorphous material system, such as Fe_3_O_4_/SiO_2_^10^, Co/Cu^11^ to magnetic/organic material system such as Fe_3_O_4_/oleic acid^12^ nanocomposites. However, in the field of semiconductor spintronics, studies on spin dependent transport by means of core-shell structural system are rarely reported. In fact, magnetic fluorescent bi-functional Fe_3_O_4_/ZnS nanocomposites with core shell structure would be an appropriate system for semiconductor spintronic studies. Similar to zinc oxide, zinc sulfide is also an II-VI compound semiconductor with large and tunable bandgap, while the former has been extensively studied as a promising magnetic semiconductor for spintronic devices^13^,^14^. Nonetheless, previous studies were mostly focused on the photoluminescence properties because it is a prominent fluorescent material, and few researches on the spin dependent transport was reported. One important reason is that the direct deposition crystalline semiconductor on magnetic core to form a well-defined core shell structure is still a challenging issue, due to the large lattice mismatch between semiconductor nanocrystals and magnetic cores^15^.

In this paper we demonstrate that seed mediate growth method can be successfully employed for the deposition of high quality crystalline ZnS shell with controllable thickness on Fe_3_O_4_ particles to form a well-defined core shell structure. With varying the shell thickness of Fe_3_O_4_/ZnS nanocomposites we investigate the spin dependence of transport behavior in II–VI compound semiconductor ZnS. Our results demonstrate that the existence of spin injection process from Fe_3_O_4_ core to ZnS coating layer which effectively reduces the spin scattering between Fe_3_O_4_ particles, and gives rise to an enhanced magnetoresistance (MR) ratio.

## Results

**Figure 1:**
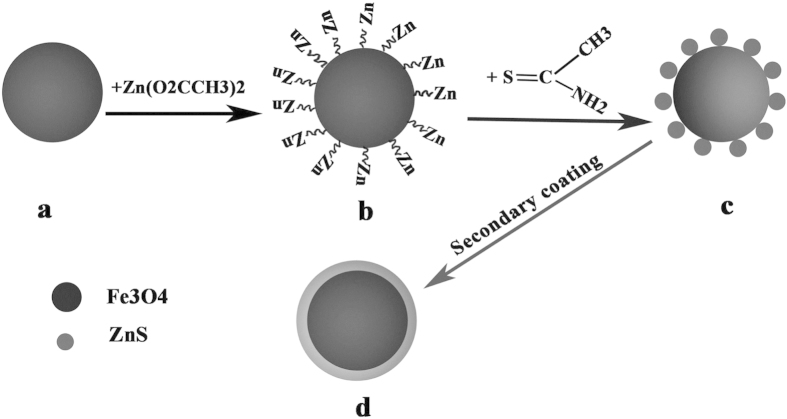


Figure 1 shows the schematic illustration of the formation mechanism of bifunctional magnetic fluorescence Fe_3_O_4_/ZnS core-shell nanoparticles: Fe_3_O_4_ particles were well dispersed in Zinc ion-rich solution after ultrasonication, then the added thioacetamide (TAA) directly reacted with the Zn ions attached on the surface of Fe_3_O_4_ particles, and ZnS particles were produced according to the following reaction equation:


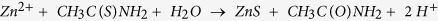


Although the ZnS nanocrystals around the Fe_3_O_4_ cores does not directly form a shell layer due to the lattice mismatch, they acts like seeding layer which made the surface of magnetic nanoparticles more “ZnS-philic”, and give rise to the formation of crystalline ZnS layer on the surface of Fe_3_O_4_ in the identical second coating process. With this approach Fe_3_O_4_/ZnS core shell nanocomposites were fabricated successfully, and the shell thickness can be well controlled by altering the elongation of coating process.

**Figure 2:**
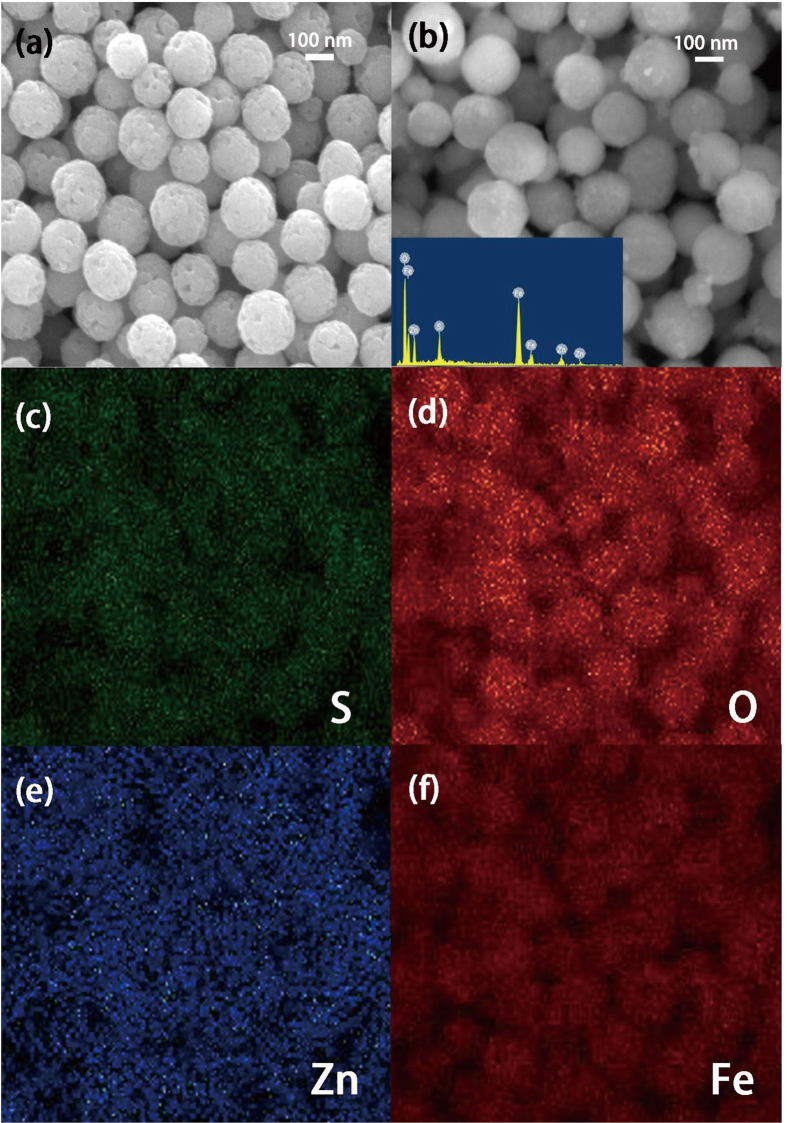
(**a**) SEM image of Fe_3_O_4_ particles. (**b**) SEM image and EDS pattern (the inset) of Fe_3_O_4_/10 nm ZnS nanocomposites. (**c**)–(**f**) EDS elemental mappings of Fe_3_O_4_/10 nm ZnS nanocomposites.

Scanning electron microscope (SEM) and energy dispersive spectroscopy (EDS) analysis were performed for the morphology and composition analysis of as prepared samples. SEM image of Fig. 2a reveals the rough surface of Fe_3_O_4_ particles, while in Fig. 2b, due to the coating of fine ZnS shell nanocrystals, relatively smooth surface is observed for Fe_3_O_4_/ZnS nanocomposites. The composition of Fe_3_O_4_/ZnS core-shell particles were measured by EDS, and the elements detected are Fe, O, Zn and S only, as shown in the inset. The EDS mappings of above elements are also presented in Fig. 2c–f, Fe and O elements are found to be located in the core area and surrounded by Zn and S elements, which imply the core-shell structure of our Fe_3_O_4_/ZnS nanocomposites.

**Figure 3:**
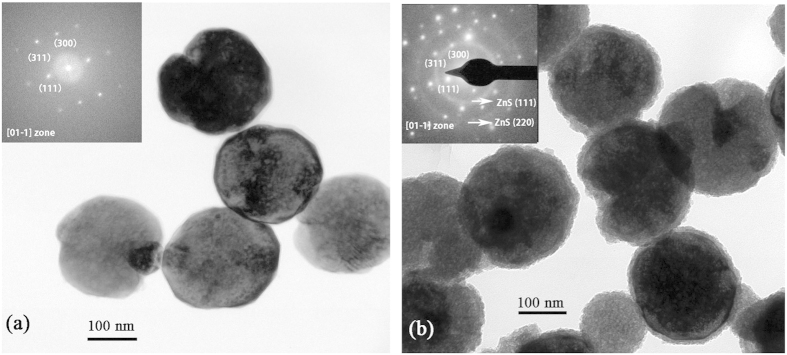
TEM images of as prepared Fe3O4 particle (**a**) and Fe_3_O_4_/10 nm ZnS nanocomposites (**b**), the insets show their corresponding SAED patterns.

In order to further confirm the core-shell structure of Fe_3_O_4_/ZnS nanocomposites, transmission electron microscope (TEM) characterization was performed, as shown in Fig. 3. In Fig. 3a the average diameter of spherical Fe_3_O_4_ particles is found to be spherical with diameters of about 200 nm. The corresponding selected area electron diffraction (SAED) pattern, shown in the inset, reveals the nearly single crystalline nature Fe_3_O_4_ phase with a [01–1] zone axis. After being coated by ZnS, the diameters of particles increase to around 210 nm (Fig. 3b), and obvious difference in contrast between the central part and the fringe is observed, which confirms the core-shell structure of our sample. The corresponding SAED pattern (the inset) can be assigned to the superposition of polycrystalline ZnS and nearly single crystalline Fe_3_O_4_, in which the diffraction rings are originated from the zinc blende phase of ZnS (111) and (220) planes.

**Figure 4:**
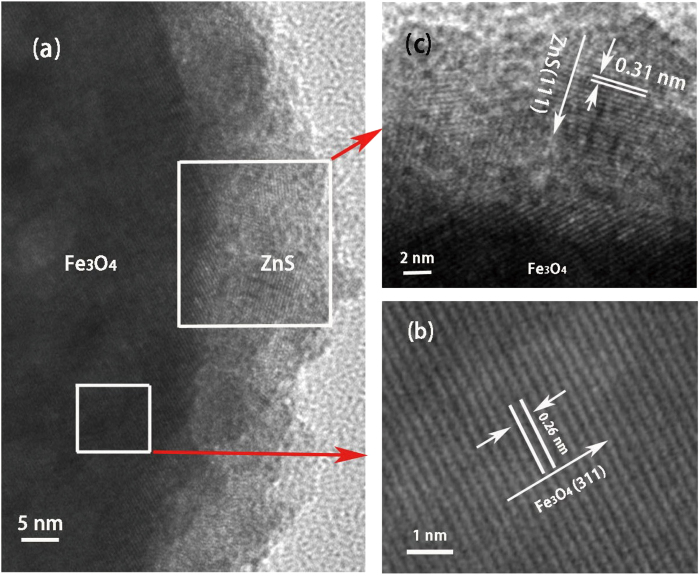
(**a**) HRTEM image of Fe_3_O_4_/10 nm ZnS nanocomposite. (**b**) and (**c**) are the lattice fringes of Fe_3_O_4_ and ZnS respectively.

High resolution TEM (HRTEM) analysis provides more detailed structure information of Fe_3_O_4_/10 nm ZnS nanocomposites. The interface image shown in Fig. 4a confirms the direct deposition of crystalline ZnS layer on the surface of Fe_3_O_4_ particle, and no amorphous ZnS or other interlayer component is observed. The streak image in Fig. 4b reveals the (311) orientation of the well crystalline Fe_3_O_4_ core, and the *d* spacing is measured to be 0.26 nm, which agrees well with that of standard magnetite^16^. Another *d* spacing of 0.31 nm is measured in the shell layer, as shown in Fig. 4c, which is consistent well with the *d* value of the ZnS (111) planes^17^.

**Figure 5:**
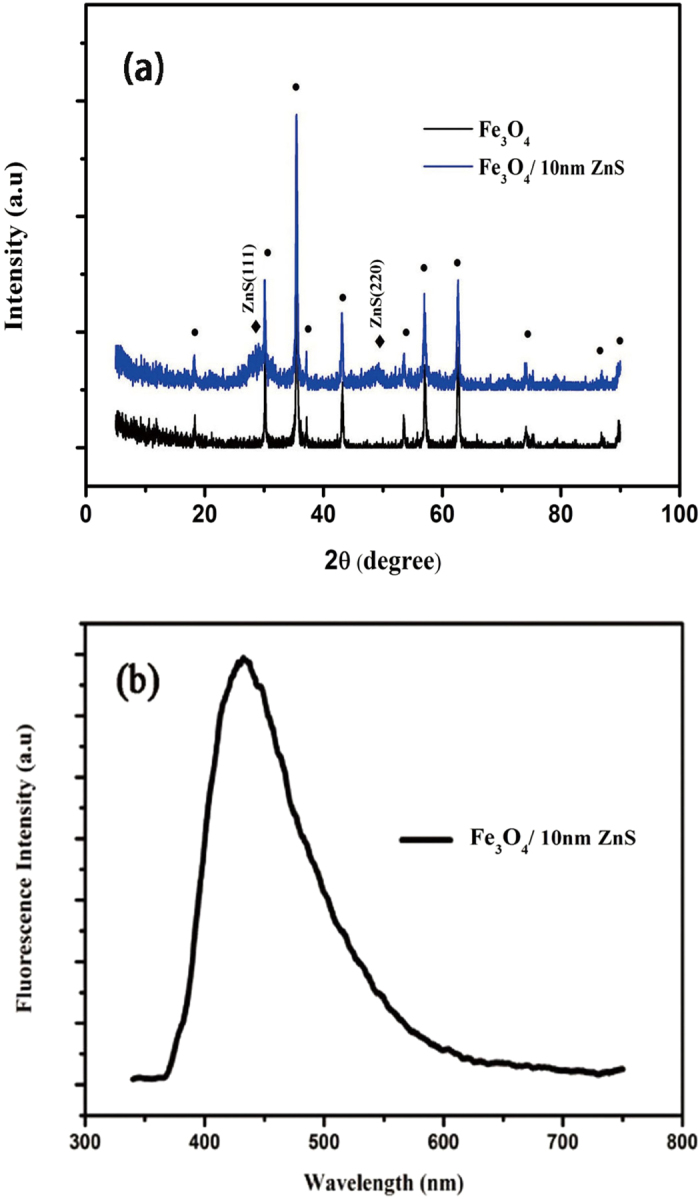
(**a**) XRD patterns of as prepared Fe_3_O_4_ and Fe_3_O_4_/10 nm ZnS particles, Symbol • and ® represent the diffraction peaks of Fe_3_O_4_ and ZnS, respectively. (**b**) Photoluminescence spectrum of Fe_3_O_4_/10 nm ZnS nanocomposites.

Fe_3_O_4_/10nm ZnS nanocomposites were further characterized by X-ray diffraction (XRD) as shown in Fig. 5a. From Fig. 5a, an inverse spinel structure is observed and additional two diffraction peaks are detected at 2θ ≈ 29.2° and 48.8° in the XRD patterns, which are corresponding to the ZnS (110) and (220) planes. The broad diffraction peaks indicate the small size of ZnS nanocrystals. Moreover, the calculated lattice parameters for Fe_3_O_4_ and ZnS are 8.377 Å and 5.306 Å, which are both close to their bulk values (8.396 Å for magnetite Fe_3_O_4_^16^ and 5.406 Å for sphalerite ZnS^17^). The luminescence property of the samples are investigated by photoluminescence (PL) spectrums, as shown in Fig. 5b .The PL spectrum shows the coated nanocomposites have a broaden emission band between 370 nm and 600 nm with emission maximum around 430 nm when excited by 330 nm light, which agrees well with defect emission in pure ZnS crystal^18^. The donor-acceptor pair transition might contribute to the blue emission of ZnS nanocrystalline^19^,^20^, in which the acceptor related to the Zn^2+^ vacancy is considered.

**Figure 6:**
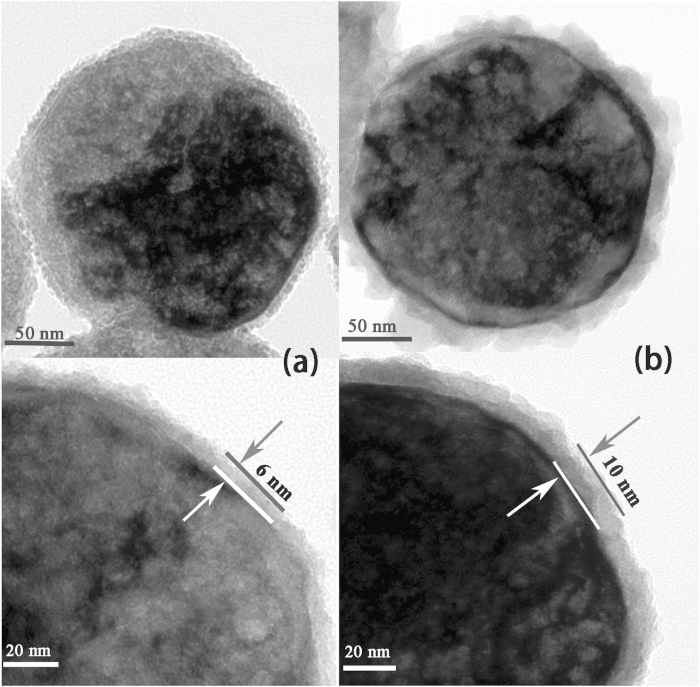
TEM images of Fe_3_O_4_/ZnS nanocomposites with shell thicknesses of 6 nm (**a**) and 10 nm (**b**).

**Figure 7:**
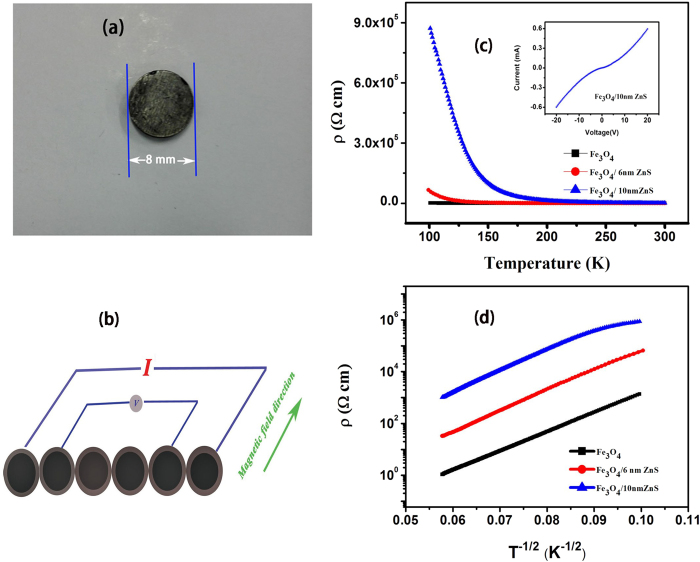
(**a**) The compact Fe_3_O_4_/ZnS nanocomposites. (**b**) The illustration of electrical and magnetic transport measurements of Fe_3_O_4_ and Fe_3_O_4_/ZnS nanocomposites. (**c**) Resistivity as a function of temperature of Fe_3_O_4_ and Fe_3_O_4_/ZnS nanocomposites. (**d**) log
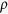
vs *T*^−1/2^ relation of Fe_3_O_4_ and Fe_3_O_4_/ZnS nanocomposites.

To study the spin transport behavior in ZnS, Fe_3_O_4_/ZnS nanocomposites with thinner shell thickness (6 nm) were also synthesized, as shown in Fig. 6a. For comparison, we also present the TEM image of Fe_3_O_4_/10 nm ZnS nanocomposites in Fig. 6b. All the prepared samples were cold pressed into pellets (8 mm in diameter, Fig. 7a) under pressure of 5 MPa for magnetism and transportation studies.

Measurements of magneto-electrical transport were performed with four-probe configuration, as shown in Fig. 7b, and the temperature dependences of resistivity (ρ-T) for Fe_3_O_4_, Fe_3_O_4_/6nm ZnS and Fe_3_O_4_/10 nm ZnS are showing in Fig. 7c. The resistivity of pure Fe_3_O_4_ pellet is around 1.08 Ω cm at room temperature, which is almost 3 orders of magnitude higher than the bulk magnetite resistivity of 5 × 10^−3^ Ω cm^21^. The extraordinary large resistivity indicates that the resistance mainly arises from the electrons scattering or tunneling at the interface between each Fe_3_O_4_ particles, which cause higher barrier than that of grain boundary in bulk material. As the dominating interface conduction mechanism, i.e., resistance inside particles makes little contribution in system conduction, no resistivity transition is observed at Verwey temperature (120 K) of Fe_3_O_4_ in ρ-T curves^22^. Moreover, even larger resistivities are observed for ZnS coated samples (33.0 Ω cm for Fe_3_O_4_/6 nm ZnS and 1029.3 Ω cm for Fe_3_O_4_/10 nm ZnS), indicating the ZnS layer significantly blocks the electrons and enhances the interface barrier. The observation of nonlinear symmetric I-V characterization curve for Fe_3_O_4_/10 nm ZnS also confirms the existence of tunneling barrier in Fe_3_O_4_/ZnS nanocomposites, as shown in the inset. Figure 7d shows the resistivities plotted on a logarithmic scale *T*^−1/2^. From the figure, we see that the linear relationships are exhibited for all samples, suggesting a typical particle boundary tunneling conductance mechanism^23^,^24^.

**Figure 8:**
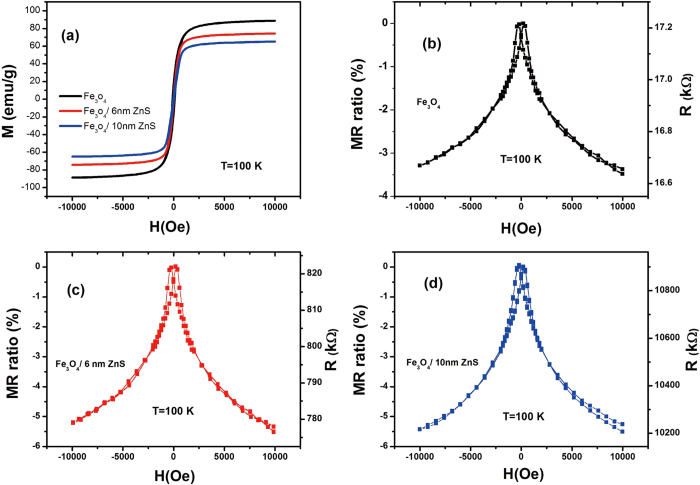
(**a**) M-H curves at 100 K. (**b**)–(**d**) MR curves measured at 100 K of pure Fe_3_O_4_, 6 nm and 10 nm ZnS coated Fe_3_O_4_, respectively.

Figure 8a shows the field dependence of magnetizations at 100 K for Fe_3_O_4_ and Fe_3_O_4_/ZnS nanocomposites. Typical hysteresis loops with saturation field of around 5000 Oe are observed for all samples. The saturation magnetization (*Ms*) is 87 emu g^−1^ for pure Fe_3_O_4_ sample, which is close to the bulk Fe_3_O_4_ value of 92 emu g^−1^ at room temperature. After the coating by ZnS, *Ms* decreased to 73 and 64 emu g^−1^ for Fe_3_O_4_/6 nm ZnS and Fe_3_O_4_/10 nm ZnS, respectively. The decrease of magnetization is reasonable as the weight fraction of ferromagnetic component is decreased in Fe_3_O_4_/ZnS nanocomposites. Nevertheless, all samples demonstrate well ferromagnetic behavior.

The field dependence of MR, defined as (*ρ*_*H*_ − *ρ*_0_)*/ρ*_0_, where *ρ*_0_ and *ρ*_*H*_ are the resistivities under zero field and applied field respectively, are measured for all samples at 100 K, as shown in Fig. 8b–d. Butterfly shapes MR curves are obtained for all samples, and no saturation trends are observed even at field of 10000 Oe although their saturation magnetization fields are only around 5000 Oe. Besides, MR-H curves do not follow the relation for granular tunneling system described as *MR* ∝ −(*M/M*_*s*_)^2^, where
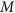
is the global magnetization and *M*_*s*_ is the saturation magnetization^24^. The phenomenon indicates that in addition to the direct tunneling, inelastic tunneling might also exist in the conduction mechanism of our samples. The conduction in inelastic tunneling is spin-independent^10^,^25^,^26^, which suppressed the spin dependent transport in the system, and could give rise to the anomalous MR behavior at low field. In addition, the remaining organic layer at the Fe_3_O_4_ surface generated during fabrication process would affect the electron transport among the particles, which may also contribute to the unusual MR behaviors. For the high field, the resistivity is gradually decreased, giving rise to linear increased MR. The linear increased MR behavior at high field is commonly observed in half-metallic compact powder and polycrystalline films^10^,^21^,^26^ , which is arising from the local spins in the grain boundaries. Generally, the high field MR is related with the magnetic susceptibility χ_gb_ of the grain boundary region^27^.

The ZnS coated Fe_3_O_4_ samples show higher MR ratio of −5.5% in an applied field of 10000 Oe while the MR ratio for pure Fe_3_O_4_ is only −3.6%, which indicates the significant role played by ZnS shell in the enhanced MR effect, and implies the existence of spin injection process. It’s worth to note that the MR ratio is hardly affected by the thickness of ZnS layer. However, the absolute MR (*ρ*_*H*_ − *ρ*_0_) of Fe_3_O_4_/10 nm ZnS is much larger than that of Fe_3_O_4_/6 nm ZnS even by order of magnitude. The almost same MR ratio but different absolute MR of Fe_3_O_4_/ZnS with different shell thickness suggests that the additional electron scattering in the ZnS layer are different for charge and spin. Obviously, the scattering of charge carriers in the ZnS layer is increased, which gives rise to the increase in resistivity. While the spin of carriers experience spin injection, and the spins in ZnS layer injected from Fe_3_O_4_ core experience weak spin scattering due to the weak spin-orbit coupling in ZnS layer and give rise to an enhanced MR effect. The observed magnetic transport properties demonstrate that ZnS might be an appropriate candidate for semiconductor material applied in spintronics.

Figure 9 shows the temperature dependence of MR at 10000 Oe and the calculated spin polarization (*P*) of all samples. It is evident that the MR for all samples monotonically increases as decreasing temperature from 300 K to 100 K. Due to the enhancement of direct tunneling at low temperature, the spin scattering caused by inelastic tunneling is suppressed^26^, which give rise to the increased MR at low temperature. The Fe_3_O_4_ samples coated by ZnS layer with both thicknesses (6 nm and 10 nm), show larger MR than that of pure Fe_3_O_4_ in the entire temperature range and the enhancement in MR is stronger at lower temperature. By calculation of *P* from MR = *P*^2^/(1 + *P*^2^) for the granular ferromagnets^28^, the temperature dependence of spin polarization is found to be similar as that of MR. However, the deduced spin polarization of Fe_3_O_4_ at 300 K is 13.5% , which is comparable with some reported experiments^10^,^29^, but still far below the expected value of half metallic Fe_3_O_4_ (100%), especially at room temperature. The deterioration of spin polarization is partly due to the unsaturated MR effect even at 10 kOe, in addition, inelastic-tunneling at grain boundaries is also suggested to be responsible for the spin polarization loss^26^,^30^. The almost same spin polarization of Fe_3_O_4_ coated by ZnS with different thickness suggests the spin coherence length^31^,^32^ in ZnS is longer than 10 nm. Further experiments are needed for the determination of spin coherence length in ZnS, such as the study of MR behavior in Fe_3_O_4_ with thicker ZnS coating layer etc.

## Discussion

To overcome the challenge of synthesizing crystalline nanocomposites with core-shell structure, various methods have been proposed. For instance, amorphous silica and carbon were utilized as interlayers to synthesize well defined core shell structural Fe_3_O_4_@SiO_2_@CdS^33^ and Fe_3_O_4_@C@TiO_2_^34^ spheres. However, the inserted buffer/interlayer usually complicates the synthetic procedures and could introduce unnecessary drawback. Another method proposed by Yu *et al.*^15^ is using anion surfactant to modify the surface of Fe_3_O_4_ particles, which achieves the direct coating of ZnS nanocrystals on surface modified Fe_3_O_4_ core. Although no other component was introduced as interlayer in this method, amorphous ZnS layer still exists between crystalline core and shell.

In this letter, we demonstrate that well defined crystalline core-shell structural Fe_3_O_4_/ZnS nanocomposites can be obtained by seed mediate method, and the shell thickness can be easily controlled by altering the elongation of coating process, which provide a promising system for spintronic studies on semiconductors.

II-VI group composite semiconductor such as ZnO has been verified as a good candidate for spintronic material, in which spin-dependent magnetic phenomena can be manipulated. As another II–VI group composite semiconductor, ZnS might also be a proper candidate in spin injection devices, in consideration of its large (3.7 ev) and tunable band gap. In fact, an enhanced MR effect is indeed observed in our Fe_3_O_4_/ZnS samples, which indicates that in addition to traditional applications, Fe_3_O_4_/ZnS nanocomposites could also be applied in the field of spintronics.

## Conclusion

In summary, we demonstrate that core shell structural Fe_3_O_4_/ZnS bi-functional nanocomposites can be well synthesized by a facile seed mediate growth method. Morphological and structural characterizations reveal the well-defined phases and high crystalline quality of Fe_3_O_4_ core and ZnS shell. The spin dependent transport studies are performed on Fe_3_O_4_, Fe_3_O_4_/6nm ZnS and Fe_3_O_4_/10nm ZnS compact powder, in which an enhanced MR effect is observed on ZnS coated Fe_3_O_4_ nanocomposites. In addition, it is experimentally demonstrated that the charge and spin of conduction electrons experience different scattering effect in ZnS layer, giving rise to two mechanism of conduction for charge and spin dependence, which have desirable potential application in spintronics.

## Method

### Materials

Ferric chloride hexahydrate (FeCl_3_·6H_2_O), sodium acetate (NaAc), zinc acetate dihydrate (Zn(Ac)_2_·2H_2_O), ethylene glycol (EG), polyethylene glycol (PEG, *M*_*w*_ = 2000) and thioacetamide (TAA, C_2_H_5_NS) are obtained from Sinopharm Chemical Reagent Co., Ltd (Shanghai, China). All chemicals in this experiment are of analytical grade and used as received without further purification. Deionized water is used throughout.

### Synthesis of monodisperse Fe_3_O_4_ nanoparticle

The monodispersed Fe_3_O_4_ nanospheres were synthesized using a solvothermal reduction method^16^ as follows : FeCl_3_·6H_2_O (1.35 g, 5 mmol) was dissolved in 40 ml EG solution to form a clear solution, followed by the addition of NaAc (3.6 g) and polyethylene glycol (1.0 g). The mixture was stirred vigorously for 30 min and then sealed in a teflon lined stainless-steel autoclave (50 mL capacity). The autoclave was heated and maintained at 200 °C for 8 h, then allowed to cool to room temperature. The black products were rewashed several times with ethanol and water and dried at 60 °C for 5 h.

### Synthesis of Fe_3_O_4_/ZnS core shell nanocomposites with shell thickness of 6 nm

1^st^ coating: 0.016 g Fe_3_O_4_ nanoparticles were well dispersed in 250 ml beaker within 200 ml aqueous solution. Mechanical stirrer and ultrasonic cleaner were employed to favor the dispersion process. After stirring and ultrasonic vibrating for 30 min, 2 mmol Zn(Ac)_2_·2H_2_O and 20 ml 0.2 mol l^−1^ TAA solution were added in the well dispersed Fe_3_O_4_ solution sequentially, and then the mixture was vigorously stirred and ultrasonic vibrated for 2 h. The products were collected and washed by water and ethanol with the help of an external magnetic force, afterwards the coated particles were transferred to vacuum oven and heated at 60° C for 5 h.

2^nd^ coating: The above coated Fe_3_O_4_ particles were redispersed in 200 ml aqueous solution, followed by the addition of ZnAc and TAA sequentially, and then the mixture was vigorously stirred and ultrasonic vibrated for 2 h. The amount of all the raw materials were chosen according to the 1^st^ coating process and the reaction was performed under the same condition as used in 1^st^ coating process.

### Synthesis of Fe_3_O_4_/ZnS core shell nanocomposites with shell thickness of 10 nm

The above Fe_3_O_4_/ZnS core shell nanocomposites with 6 nm shell thickness were redispersed in 200 ml aqueous solution, followed by the addition of ZnAc and TAA sequentially, and then the mixture was vigorously stirred and ultrasonic vibrated for 2 h. The amount of all the raw materials were chosen according to the 1^st^ coating process and the reaction was performed under the same condition as used in1^st^ coating process.

### Characterization

The TEM and HRTEM pictures were obtained with a Tecnai G2 20 200 kV transmission electron microscope. The samples’ crystallographic and structural characterizations were investigated by Rigaku Smartlab 3 X-ray diffractometer with Cu Kα radiation (*λ* = 1.5418). The magnetic and luminescence properties were studied by a Lakeshore7407 vibrating sample magnetometer and photoluminescence spectrophotometer (Horiba Jobin Yvon, Fluorolog-3), respectively. The electrical transport measurements were performed on a home-made physical property measurement system.

## Additional Information

**How to cite this article**: Liu, E. *et al.* Investigation on Spin Dependent Transport Properties of Core-Shell Structural Fe^3^O^4^/ZnS Nanocomposites for Spintronic Application. *Sci. Rep.* **5**, 11164; doi: 10.1038/srep11164 (2015).

## Figures and Tables

**Figure 9:**